# Automated screening for clinically ascertained loss of cerebral functions in patients with severe brain injury—study protocol for a cluster-randomized interventional trial

**DOI:** 10.1186/s13063-025-09354-z

**Published:** 2025-12-11

**Authors:** Daniela Schoene, Martin Roessler, Konrad Pleul, Sepp Hoehne, Anne Trabitzsch, Daniela Barnett, Andreas Guenzel, Hagen B. Huttner, Martin Sedlmayr, Albrecht Günther, Kristian Barlinn, DETECT-IVE Investigators

**Affiliations:** 1 Department of Neurology, Faculty of Medicine, University Hospital Carl Gustav Carus, Technische Universität Dresden, 01307 Dresden, Germany; 2 BARMER Institute for Health Care System Research (Bifg), Berlin, Germany; 3 Deutsche Stiftung Organtransplantation (DSO), Frankfurt am Main, Germany; 4 Institute for Medical Informatics and Biometry, Faculty of Medicine, University Hospital Carl Gustav Carus, TUD Dresden University of Technology, Dresden, Germany; 5 Faculty of Medicine, University Hospital Carl Gustav Carus, Technische Universität Dresden, Dresden, Germany; 6 Department of Neurology, University Hospital Jena, Jena, Germany

**Keywords:** Organ donation, Brain death, Death by neurologic criteria, Detection, Screening, Brain injury

## Abstract

**Background:**

The low organ donation rate in Germany is associated with deficiencies in the identification of patients at risk of developing brain death. An automated digital clinical support system (DETECT) was designed to prospectively identify intensive care patients who are at risk of developing brain death. The objective of the study is to evaluate the effectiveness of DETECT in the detection of patients with severe brain injury who progress toward brain death compared to standard practice without DETECT.

**Methods:**

The study will follow a multicentre, cluster-randomized, controlled design conducted at 19 sites in Germany over a 30-month period. The study will include patients aged ≥ 18 years with primary or secondary acute brain injury, requiring mechanical ventilation and death during hospital stay. DETECT periodically processes real-time data from the ICU information system to screen for a combination of coma and absent bilateral pupillary light reflexes—both considered early indicators of impending brain death. In the event of a positive screen, an automated notification will be sent to the designated transplant coordinators. The email is intended to prompt clinical assessment of the detected patient and, if necessary, initiate a guideline-based brain death evaluation. The primary endpoint is the identification of patients who develop brain death during hospitalization. Secondary endpoints encompass missed identification of potential brain death cases and deceased organ donations. Upon completion of the study, a survey will be conducted to investigate the stakeholders’ experiences with DETECT.

**Discussion:**

Findings will provide insights into the effectiveness of an automated digital support system for the detection of patients at risk of developing brain death and potential organ donors. Automation may enhance ICU workflow efficiency and timely decision-making in organ donation processes.

**Trial registration:**

ClinicalTrials.gov NCT06293170. Registered on March 5, 2024

**Supplementary Information:**

The online version contains supplementary material available at 10.1186/s13063-025-09354-z.

## Background

### Organ donation in Germany

Organ donation is often the only chance to improve or even save the lives of critically ill patients with end-stage organ failure. However, Germany has struggled with persistently low organ donation rates for many years. In 2023, the country recorded a rate of 11.4 organ donors per million inhabitants—one of the lowest rates in Europe [1]. In contrast, Spain recorded 46 organ donations per million inhabitants in 2022 [2]. In addition, many patients do not receive a transplant in time because of long waiting times for donor organs in Germany. In 2022, of approximately 8500 patients on Germany’s transplant waiting list, 743 died before receiving an organ [2]. A large-scale secondary data analysis of 112 million hospital admissions suggests that one key factor behind the persistently low organ donation rate is the inadequate identification of potential organ donors in hospitals [3]. While the number of potential organ donors in Germany increased by 13.9% from 2010 to 2015, actual organ donations declined by 32.3%. Another state-wide analysis of health data in Germany further highlighted deficiencies in the identification processes [4]. In 2016, brain death evaluation was not initiated in 3.4% of 7389 cases, even though retrospective analysis indicated that it would have been clinically warranted. Since brain death is the medical prerequisite for deceased organ donation in Germany, failure to identify patients who potentially progress toward brain death also means a failure to identify potential organ donors. The early identification of such patients requires advanced expertise in neurocritical care [5]. However, such expertise is often lacking, particularly in non-neurological critical care fields such as anaesthesia, surgery, and internal medicine, where patients with acute brain injury are nonetheless frequently treated due to the limited availability of neurocritical care.

### Determination of brain death

In Germany, deceased organ donation requires the determination of brain death, which is defined as the complete and irreversible cessation of all cerebral and brainstem functions [2, 6]. The diagnostic criteria for brain death are regulated under Section 16 of the German Transplantation Act (TPG) and the guidelines for brain death determination by the German Medical Association (*Bundesaerztekammer*, BÄK) [7, 8]. These guidelines are legally binding and are widely considered both scientifically and medically uncontroversial [6]. The diagnosis of brain death requires the presence of a severe acute primary and/or secondary brain injury. A standardized neurological examination requires the documentation of coma, the absence of all brainstem reflexes, and apnoea. The irreversibility of these findings must be confirmed either through repeated neurological examinations over a defined observation period or by ancillary tests, such as CT angiography or transcranial Doppler to confirm circulatory arrest or electroencephalography to confirm electrocerebral inactivity [9]. A neurologist or neurosurgeon with substantial expertise in neurocritical care must be involved in the assessment. However, access to neurocritical care specialists with expertise in brain death determination is limited to a small number of hospitals in Germany and is barely available in rural hospitals despite having general intensive care unit capabilities. In such cases, brain death determination is conducted by a neurological or neurosurgical consultation service guided by the German organ procurement organization (*Deutsche Stiftung Organtransplantation*—DSO). The utilization of this service, however, requires initial identification of suspected cases progressing toward brain death.

### Digital screening for early detection of impending brain death

**Fig. 1:**
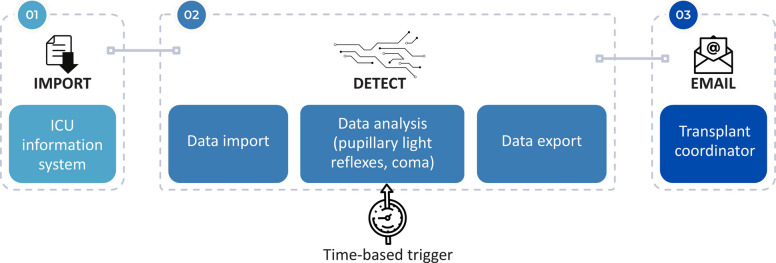
Data processing in DETECT

DETECT (*AutomateD ScrEening for Clinically AscerTainEd Loss of Cerebral FuncTions in Patients with Severe Brain Injury*) is an automated digital clinical support system that was initiated at the University Hospital Carl Gustav Carus Dresden and further developed in collaboration with the Data Integration Center (DIZ) of University Hospital Carl Gustav Carus Dresden and the DSO. DETECT periodically screens data entered manually into the ICU information system according to defined criteria and generates a list of detected cases with potentially impending brain death. The defined criteria for screening include clinical findings such as coma, categorized as a Richmond Agitation Sedation Scale (RASS) score of −4 or −5 or a Glasgow Coma Scale (GCS) score of 3 to 5, and the bilateral absence of pupillary light reflexes, entered as “present” or “absent”. These parameters are considered predictive of impending brain death in patients with acute brain injury and are routinely assessed several times a day by intensive care unit physicians and nursing staff. Further findings provided such as serum sodium level, ventilation mode, intracranial pressure (ICP), cerebral perfusion pressure (CPP), and previous resuscitation status are not primarily used by DETECT for direct assessment of the patient’s condition but provide the hospital’s transplant coordinators with additional clinical information. In the event of positive detection, a notification is sent automatically via the hospital’s internal email server to transplant coordinators or another designated group of recipients (Fig. 1). This notification includes above-mentioned patient data and is intended to support standardized processes comprising individual case evaluation by intensive care physicians. If the constellation of findings is confirmed, it may lead to brain death evaluation.

DETECT was prospectively validated in 414 hospitalized patients with acute brain injury, where it demonstrated a sensitivity of 100% and a specificity of 89% for identifying patients in whom brain death was subsequently confirmed [10]. A retrospective cohort study investigated the impact of DETECT on missed brain death cases [11]. After the implementation of DETECT at a tertiary care hospital, the rate of missed brain death cases dropped from 8.5% to 0.7%, representing a 93% relative risk reduction (*OR* 0.07, 95% *CI* 0.01–0.57). In contrast, the control group comprising tertiary care hospitals without DETECT support showed no significant change in the relative risk (*OR* 1.11, 95% *CI* 0.66–1.88, *p* = 0.002) [11].

### Study objectives

The DETECT-IVE trial aims to provide causal evidence for the effectiveness of DETECT in increasing brain death detection rates. Secondary objectives of the trial are to investigate the relationship between the implementation of DETECT and (1) missed cases with potentially impending brain death, (2) deceased organ donations following brain death, and (3) interactions with the German organ procurement organization to assess various aspects of the implementation and effectiveness of DETECT. In addition, a survey will be conducted among intensivists at participating hospitals to assess their experience with the implementation of DETECT. This will help to assess integration into existing clinical workflows, identify potential challenges, and identify opportunities for improvement.

### Hypotheses

The primary hypothesis is that the implementation of DETECT causally increases the probability of detecting patients with brain death in hospitals without prior use of DETECT.

Secondary hypotheses are that the implementation of DETECT is related to the following:

- A lower probability of missing patients with potentially impending brain death
- A higher probability of deceased organ donation
- A higher probability of donation-related interactions with the organ procurement organization in hospitals without prior use of DETECT

## Methods/design

### Study design

**Fig. 2:**
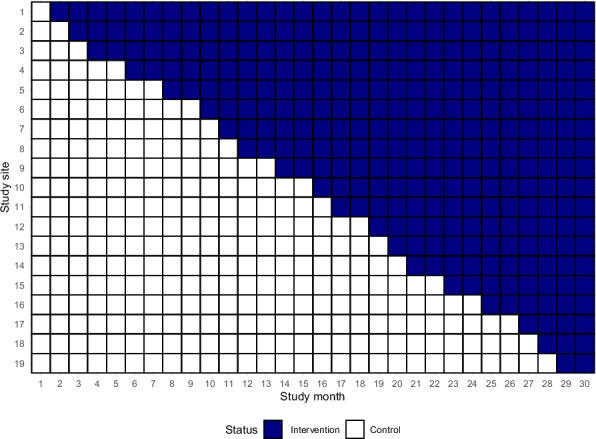
Stepped-wedge design

The effectiveness of DETECT will be investigated by using a cluster-randomized controlled trial in a stepped-wedge design (SW-RCT) [12]. The stepped-wedge approach allows causal inferences about the effect of the intervention, similar to conventional randomized controlled trials. However, it has the advantage that all participating hospitals will implement DETECT during the study period, ensuring no hospital is excluded from the intervention. The intervention phases will be distributed over a 30-month observation period, with each hospital initially included under standard care conditions (control phase). The stepwise implementation of DETECT will then follow in a randomized sequence, as shown in Fig. 2. The allocation of hospitals to specific intervention timepoints will be randomized using a computer-generated randomization process and conducted by the study statistician before the start of the study. After entering the intervention phase, each hospital is expected to continuously use DETECT throughout the rest of the study period (Table 1). More details on the trial design and the randomization process are provided in the statistical analysis plan (SAP) (see supplementary material). The study protocol was prepared following the SPIRIT 2013 guidelines (Standard Protocol Items: Recommendations for Interventional Trials) [13].

**Table 1:** SPIRIT flow diagram for the DETECT-IVE trial—a stepped-wedge cluster-randomized trial

			**Study period**					**Close-out**
	**Site selection**	**Site allocation**	**Control**	**Intervention implementation**				**Post-implementation**
**Timepoints**	Pre-trial initiation		Step 0	Step 1	Step 2	…	Step 19	t x
Eligibility screen	X							
**Randomization**		X						
**Technical readiness check**		X						
**Interventions**								
**DETECT pre-implementation meeting**			→	→	→	→	→	
**DETECT start**				→	→	→	→	
**Assessments**								
**Data collection (TransplantCheck)**			→	→	→	→	→	
Survey								X
**Data analysis**								X

## Trial organization, governance, and monitoring

The coordinating centre at the University Hospital Carl Gustav Carus Dresden is responsible for the overall management of the DETECT-IVE trial, including communication with participating sites, data management, and ensuring compliance with ethical and regulatory requirements. A Project Management Group, comprising the principal investigator, study statistician, and representatives from data management, informatics, and clinical coordination, meets monthly to review trial progress, data integrity, and adherence to the protocol. The Trial Steering Committee, including the principal investigator and senior clinical investigators from participating hospitals, provides strategic and ethical oversight, reviews study conduct and operational issues, and ensures that the trial meets its scientific and regulatory objectives. The German Organ Procurement Organization (DSO) collaborates with the coordinating centre by providing anonymized registry data in accordance with Section 9a of the German Transplantation Act (TPG) and by supporting data validation and harmonization procedures across sites.

Given the low-risk, system-level nature of the DETECT-IVE intervention, no independent Data Monitoring Committee was established.

## Patient and public involvement

Patients and members of the public were not directly involved in the design, recruitment, or conduct of the DETECT-IVE trial, as the study evaluates a system-level digital intervention focusing on clinical detection processes rather than individual patient outcomes.

## Participating hospitals

**Fig. 3:**
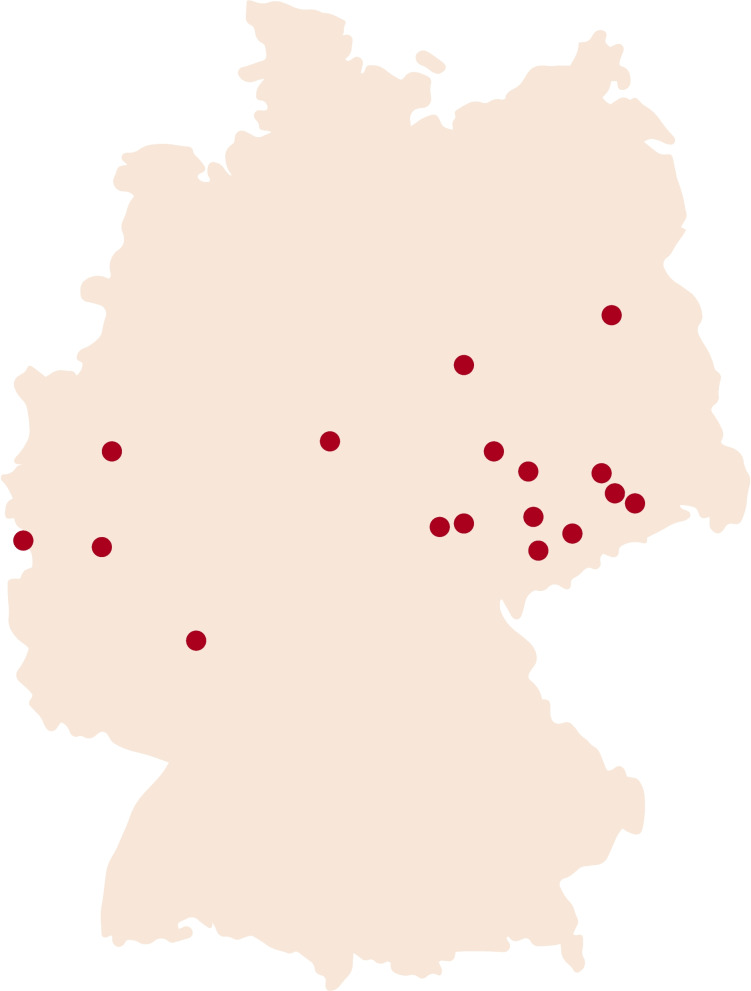
Study sites in Germany

**Fig. 4:**
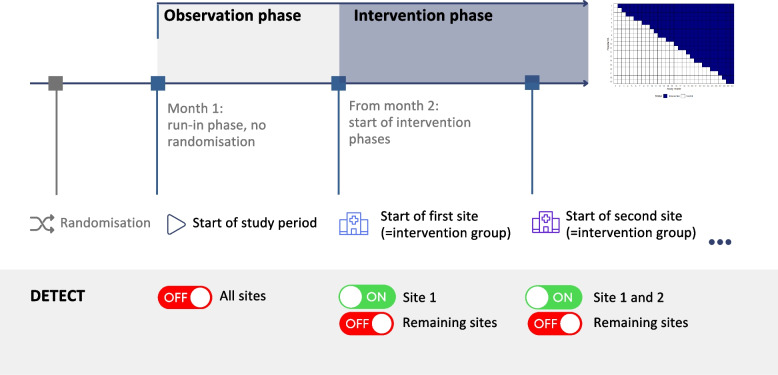
Stepped-wedge design illustrates the stepwise rollout of the intervention across study sites

In total, 19 hospitals located in different German regions were included in the study (Fig. 3). Each of these hospitals had at least one ICU treating patients potentially fulfilling the inclusion criteria at the start of the study. DETECT was not previously used at the participating hospitals but was planned for implementation. The study was designed to accompany this process and enable systematic evaluation. The intervention will roll out stepwise across 19 hospitals. Each site will switch from control to intervention at a set timepoint (Fig. 4).

## Blinding

Study physicians involved in secondary outcome assessment (i.e. potentially missed cases with impending brain death—see “Data”) are blinded to DETECT screening results.

### Data

The analysis will be based on anonymized patient data routinely collected according to the German TPG Section 9a Abs. 2. No manual data entry by each study site is required. Access will be restricted to authorized members of the study team, and data will be stored on secure, access-controlled servers. These data contain all patients with primary or secondary brain injury who died in the respective hospitals. Data include information on age, sex, type of brain injury (primary/secondary), ventilation status and duration, length of stay (total and on ICU), diagnoses coded according to the International Statistical Classification of Diseases and Related Health Problems, 10th revision, German Modification (ICD-10-GM), detected brain death, and deceased organ donation. Complete reporting of this data to the organ procurement organization is mandated by law for German hospitals. In addition, transplant coordinators in the hospitals are obliged to evaluate patient data for missed patients with potentially impending brain death using the software “DSO-TransplantCheck 4” on a regular basis [14]. The results of these retrospective evaluations are used to operationalize the number of these cases. As part of the DETECT-IVE trial, a study physician—blinded to the DETECT screening results—will accompany evaluations at all participating hospitals to ensure consistent procedures and comparable outcomes.

Patient-specific data will be complemented by information on the use of DETECT after implementation in the participating hospitals. This includes functionality, recording, and documentation of clinical findings, generation of e-mails, and time stamps. This data will be used within the framework of sensitivity analysis to explore potential heterogeneity in the effects of DETECT on the primary and secondary outcomes.

### Inclusion and exclusion criteria

The study population encompasses all patients who potentially progress toward brain death. This includes patients as follows:

- With primary and/or secondary brain injury
- That were invasively ventilated during their hospital stay
- And were discharged because of in-hospital death

Patients under 18 years of age at the time of hospital admission will be excluded.

### Outcomes

#### Primary outcome

The primary outcome of the study is the detection of patients with brain death. The definition of brain death adheres to the corresponding guideline of the German Medical Association [8]. Detection of brain death is recorded in the data provided by the hospitals to DSO according to TPG (see section “Data”).

#### Secondary outcomes

We consider the following secondary outcomes, which may be related to the use of DETECT:

- Missed case with potentially impending brain death
- Deceased organ donation
- Donation-related interaction with the organ procurement organization
- Number of alerts sent by DETECT

### Intervention

The intervention is the implementation and use of DETECT. A patient will be considered as having received the intervention if DETECT was implemented in the participating hospital before the patient was discharged because of in-hospital death.

### Confounders

Potential confounders at the patient level considered in sensitivity analyses include the following:

- Age (in years and in categories)
- Sex (male/female)
- Type of brain injury (primary/secondary)
- Selected diseases as coded by primary diagnoses (operationalizations are provided in the SAP; see supplementary material)

At the hospital level, potential confounders include the following:

- The number of patients fulfilling the inclusion criteria and not fulfilling the exclusion criteria within 1 year before the start of the study
- The share of detected brain death in the 1-year period before the start of the study
- University hospital status

The first two of these potential hospital-level confounders are used to operationalize the experience of a hospital in the treatment of patients belonging to the study population and in the detection of brain death.

### Intention-to-treat and per-protocol analysis

The main analysis will be based on the intention-to-treat (ITT) principle [15]. As a complementary approach, a per-protocol (PP) analysis will be applied [15] (see the SAP in the supplementary material for more details).

### Handling of missing values

If present, missing values will be addressed using multiple imputation by chained equations (MICE) [16]. As an alternative approach, complete case analysis will be additionally applied by excluding all patients with missing values in at least one variable entering the respective statistical model.

### Drop out/lost to follow-up

In general, a dropout or lost to follow-up of patients is not possible according to the definition of the study population. In the case of a hospital dropout, the main analysis will include the data provided by the hospital until the time of dropout. For sensitivity analysis, the complete data of hospitals with dropout will be removed from the sample.

### Statistical analysis

Descriptive statistics will be reported for all variables included in the statistical analyses (more details are provided in the SAP; see supplementary material). A significance level of 5% for all statistical tests will be applied. Statistical estimates of parameters will be reported with 95% confidence intervals. The main analysis of the primary outcome will be based on a generalized linear mixed model (GLMM) with a logistic link function and correction of degrees of freedom [17] (see SAP in supplementary material for more details). The analysis of secondary outcomes will be based on GLMMs analogous to the model specified for the primary outcome (see the SAP in supplementary material for more details). Sensitivity analyses will be conducted for the effect of the intervention on the primary and secondary outcomes as described below.

#### Sample size and power

Power of the statistical test for the effect of the intervention on the primary outcome (detection of brain death) will be calculated before the start of the study. This calculation utilized retrospective data from the period 2022–2023 on patients fulfilling the inclusion criteria in the hospitals participating in this study. It will be assumed that the number of included patients by hospital and month during the study period will be similar to this retrospective data. Further, the probability of brain death detection in the control phase is expected to be 6.9%, which is the proportion of detected patients with brain death in the retrospective data. Based on a previous study [11], the true intervention effect is set to four percentage points as the minimum clinically important difference. On a national scale, such an improvement would represent a substantial increase in the number of correctly identified patients with brain death and is therefore considered a meaningful and policy-relevant effect that warrants broader implementation. Given the trial design and the model specification for the primary outcome described above, these assumptions resulted in an expected power of 83.6%. More details on the power calculations are provided in the SAP (see supplementary material).

#### Adjustment for potential confounders

In sensitivity analyses, potential imbalances between control and intervention phases regarding the above-mentioned confounders will be addressed by inclusion of these confounders as regressors in the statistical models (see SAP in supplementary material for more details).

#### Potential effect modifications

Potential effect modifications will be explored with respect to the relationships between the intervention and the primary and secondary outcomes. Potential moderator variables include the following:

- The hospitals’ shares of detected patients with brain death 1 year prior to the start of this study
- University hospital status
- Different utilizations of DETECT

To account for potential heterogeneity across sites, variations in the utilization of DETECT will be examined as part of the sensitivity analyses. Utilization will be operationalized through several indicators reflecting the type and intensity of system use, such as the use of DETECT for documentation and communication of patient-specific information, the number of alerts generated, and the use of the built-in question feature that enables communication among clinical staff. More details on operationalizations and statistical modeling of effect modifications are described in the SAP (see supplementary material).

#### Interim analyses and stopping rules

No formal interim analyses or stopping rules are planned for this study. DETECT-IVE is a low-risk, system-level intervention that does not involve any patient-specific treatment or exposure. The intervention consists solely of automated screening and notification processes using routinely collected clinical data. Therefore, early termination for safety or efficacy is not applicable. However, trial progress and adherence to the protocol will be periodically reviewed by the coordinating center and the principal investigator to ensure data quality and compliance with study procedures.

#### Statistical software

For data preparation, SQL and Excel will be used. Statistical analysis will be conducted using R version 4.5.0 (R Foundation for Statistical Computing, Vienna, Austria; https://www.r-project.org/).

## Discussion

This study will be the first to evaluate an intervention in a multicentre randomized controlled trial specifically designed to improve the identification of patients meeting criteria for brain death. By integrating an automated clinical detection support system into routine workflows using routinely documented ICU data, the intervention seeks to enhance in-hospital processes and address well-known deficiencies in the identification of potential organ donors in Germany. If effective, DETECT could improve the detection of patients at risk of brain death and promote adherence to donor identification guidelines, thereby potentially increasing organ donation rates.

The implications of this study could be considerable. If a positive signal is observed, it would be the first trial-based evidence suggesting that digital decision support tools may contribute to improving a public health issue as organ donation in Germany. Importantly, such a system could help reduce dependence on local neurocritical care expertise by offering a standardized, data-driven screening approach that identifies relevant clinical patterns early—especially in hospitals without specialized neurological departments. In the future, such a system might serve as a trigger for telemedical consultation, whereby a remote neurocritical care expert could validate the findings through digital assessment tools and initiate an on-site examination according to the guidelines of the German Medical Association.

However, several practical and operational challenges must be acknowledged. Integrating DETECT across diverse ICU information systems is inherently complex due to the heterogeneity of hospital IT environments. For the purpose of this study, DETECT was designed to be compatible with a broad range of local IT infrastructures and ICU information system vendors. Nonetheless, differences in local data entry practices—especially regarding key parameters such as pupil size and level of consciousness—may influence the effectiveness of the intervention. To minimize variability, all participating sites underwent a harmonization process to align data mapping prior to study initiation. Further variability may arise in how individual sites respond to a positive DETECT result. Local differences in staffing structures and neurocritical care expertise may affect subsequent clinical workflows. To capture these variations and better understand the real-world applicability of DETECT, a post-study survey will assess usability, integration into clinical routines, and perceived value of the system. While participation in the survey is voluntary, the insights gained are expected to inform future refinements and support broader implementation of DETECT in clinical practice.

This study represents an important step toward addressing a critical gap in organ donor identification in Germany by evaluating the role of automated clinical detection support in neurocritical care. While several implementation challenges must be navigated, the DETECT trial has the potential to offer both clinical and structural benefits—by enabling earlier identification of potential organ donors, supporting clinical decision-making, and contributing to a more equitable organ donation system across hospitals of varying levels of expertise. If successful, DETECT could lay the foundation for scalable digital innovations in donor identification that are both evidence based and aligned with national ethical and clinical standards.

## Trial status

Protocol version is 3.0, dated August 08, 2024.

The study started on February 15, 2025. The first study site entered the intervention phase on March 15, 2025. Recruitment and data collection are ongoing and will be completed within 30 months.

## Supplementary Information

Additional file 1: Figure A1: Results of Monte Carlo power simulations of 100 randomly generated intervention orders. The gray bars indicate the frequency of simulated power values. The blue line marks the arithmetic. mean of the simulated power values. The red line marks the power level of 80%.

Additional file 2: SPIRIT Checklist for Trials.

Additional file 3: Statistical Analysis Plan.

## Data Availability

The data that support the findings of this study are available from DSO, but restrictions apply to the availability of these data, which were used under license for the current study and so are not publicly available. Data are, however, available from the authors upon reasonable request and with permission of the participating hospitals and DSO. Only the study team at the coordinating centre will have access to the final trial dataset. No contractual agreements restrict investigator access.

## Abbreviations

API: Application programming interface
BÄK: German Medical Association (*Bundesärztekammer**)*
CPP: Cerebral perfusion pressure
DETECT: AutomateD ScrEening for Clinically AscerTainEd Loss of Cerebral FuncTions in Patients with Severe Brain Damage
DIZ: Data Integration Centre
DSO: German Organ Transplantation Foundation (*Deutsche* *Stiftung* *Organtransplantation*)
GCS: Glasgow Coma Scale
ICD-10-GM: International Classification of Diseases, German Modification
ICP: Intracranial pressure
ICU: Intensive care unit
PDMS: Patient data management system
RASS: Richmond Agitation-Sedation Scale
SW-RCT: Stepped-wedge cluster-randomized controlled trial
TPG: German Transplantation Act (*Transplantationsgesetz*)
