# Supplementary material for: Automated screening for clinically ascertained loss of cerebral functions in patients with severe brain injury—study protocol for a cluster-randomized interventional trial
Source: Trials. 2025 Dec 11;27:39. doi: 10.1186/s13063-025-09354-z (PMC12802192; doi:10.1186/s13063-025-09354-z)
Supplement: Supplementary file 1 — Additional file 1: Figure A1: Results of Monte Carlo power simulations of 100 randomly generated intervention orders. The gray bars indicate the frequency of simulated power values. The blue line marks the arithmetic. mean of the simulated power values. The red line marks the power level of 80%. [file 13063_2025_9354_MOESM1_ESM.pdf]

Appendix of the Statistical Analysis Plan

AutomateD ScrEening for Clinically AscerTainEd Loss of Cerebral FuncTions in Patients with Severe Brain Injury – an InterVEntional Cluster Randomized Trial

(DETECT-IVE)

Version 1.0

July 10, 2025

Contents

Power calculation ... 2
Methodological approach ... 2
Data ... 2
Design and parameters ... 2
Design ... 2
Number of cases ... 2
Intervention effect ... 2
Outcome probabilities ... 3
Intra-cluster correlation ... 3
Estimation approach ... 3
Simulation ... 3
Results ... 3
References ... 4

Power calculation

Methodological approach

Power calculation was based on an assumed data generation process that matches the statistical model for the primary outcome described in the statistical analysis plan (SAP), section "Main analysis (primary outcome)". We used Monte Carlo simulation to calculate power given a pre-specified set of parameter values entering the data generation process. The derivation of these parameter values is described in the following.

Data

For power calculation, we used retrospective data from the period 2022-2023 on patients fulfilling the inclusion criteria, who were treated in the hospitals participating in the study. In total, this included 3,935 patients. Brain death was detected in 273 (6.9 %) of these patients.

Design and parameters

Design

Power calculation was based on a stepped wedge cluster randomized trial design. The length of the study period was 30 months. The intervention steps of the hospitals followed Figure 1 in the SAP.

Number of cases

For each participating hospital, we assumed that the number of patients per study month will develop according to the retrospective data over the study period. This approach ensured that 1) differences between hospitals regarding the number of patients fulfilling the inclusion criteria were properly handled in power calculation, 2) variations in the numbers of included patients over time were considered.

Intervention effect

A previous observational study [1] indicated that the proportion of detected patients with brain death increased by 7.09 percentage points in one hospital after implementation of DETECT, whereas this proportion increased by 2.42 percentage points in a control cohort of other hospitals that had not implemented DETECT. The difference-in-differences estimate of the intervention effect based on these data is 4.67 percentage points. Given this estimate, we chose a conservative approach for power calculation and assumed that the size of the true intervention effect is 4 percentage points.

Outcome probabilities

In line with the retrospective data, we assumed that the probability of brain death detection in the study will be 6.9 % in the control phase. Given the assumed intervention effect, this yielded an assumed probability of brain death detection of 10.9 % in the intervention phase.

Intra-cluster correlation

In the data generation process, intra-cluster correlation was represented by the variance σ^2 of the hospital-specific random effect α_h (see SAP, section: "Main analysis (primary outcome)"). We used the above mentioned retrospective data to estimate σ^2. Based on those estimation results, we assumed that $\sigma^2 = 0.19$ for power calculation.

Estimation approach

In line with the SAP, we used restricted pseudo-likelihood estimation of the generalized linear mixed model for the primary outcome in power calculation. We also corrected the degrees of freedom when deriving the p-value and the confidence interval of the intervention effect. More specifically, we assumed a t-distribution with 17 (=19 – 2) degrees of freedom.

Simulation

The hospitals participating in the study are expected to contribute different numbers of patients fulfilling the inclusion criteria to the analysis of the primary outcome. We accounted for these expected differences in patient numbers by using retrospective patient data of the hospitals for power calculation. However, an issue arising from those differences in patient numbers is that the power of the statistical test for the intervention effect depends on the intervention order of the hospitals [2]. To account for this issue, we randomly generated 100 possible intervention orders. We calculated the power for each of these intervention orders using 1,000 Monte Carlo iterations. The expected power of the statistical test of the intervention effect then was derived as the arithmetic mean the power values across intervention orders.

Results

The power calculation yielded an expected power of 83.6 % (Figure A1). None of the simulated intervention orders resulted in a power below 80 %. The most favorable intervention order had a power of 86.8 %.

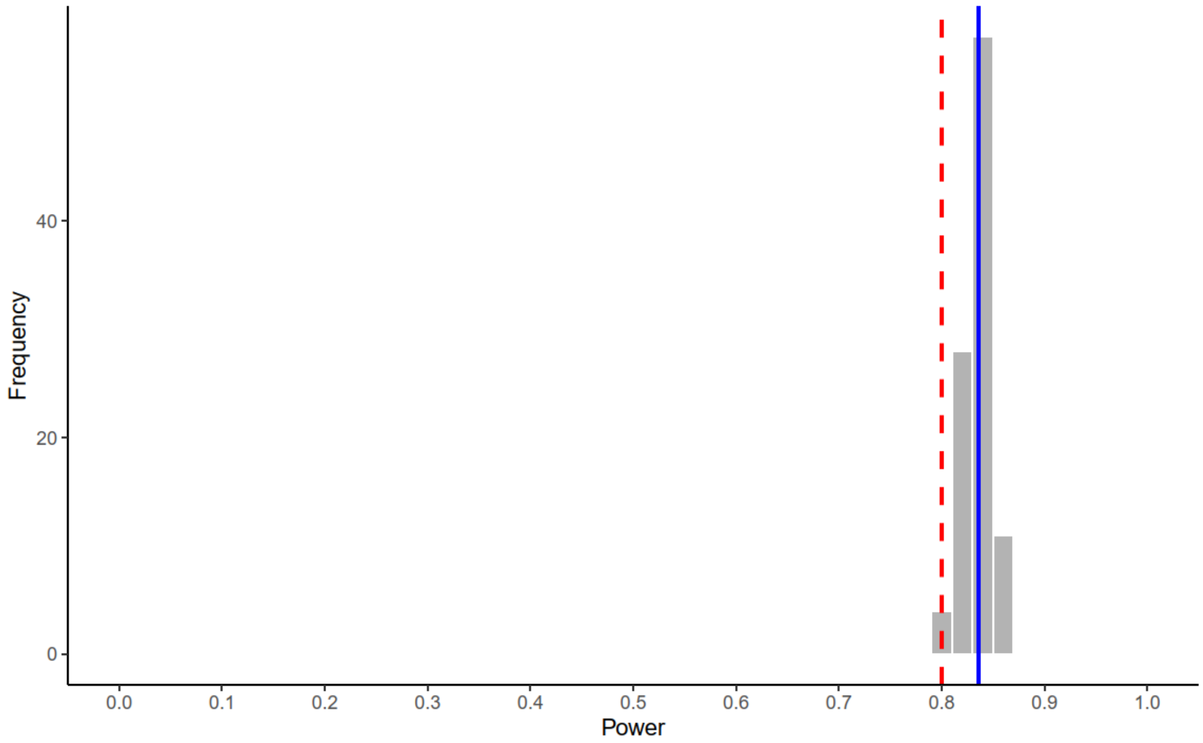

Figure A1: Results of Monte Carlo power simulations of 100 randomly generated intervention orders. The gray bars indicate the frequency of simulated power values. The blue line marks the arithmetic mean of the simulated power values. The red line marks the power level of 80 %.

References

1 Trabitzsch A, Pleul K, Barlinn K, *et al.* An Automated Electronic Screening Tool (DETECT) for the Detection of Potentially Irreversible Loss of Brain Function. *Dtsch Arztebl International*. 2021;118:683–90.

2 Martin JT, Hemming K, Girling A. The impact of varying cluster size in cross-sectional stepped-wedge cluster randomised trials. *BMC Medical Research Methodology*. 2019;19:123. doi: 10.1186/s12874-019-0760-6
