# Supplementary material for: Automated screening for clinically ascertained loss of cerebral functions in patients with severe brain injury—study protocol for a cluster-randomized interventional trial
Source: Trials. 2025 Dec 11;27:39. doi: 10.1186/s13063-025-09354-z (PMC12802192; doi:10.1186/s13063-025-09354-z)
Supplement: Supplementary file 3 — Additional file 3: Statistical Analysis Plan. [file 13063_2025_9354_MOESM3_ESM.pdf]

Statistical Analysis Plan

AutomateD ScrEening for Clinically AscerTainEd Loss of Cerebral FuncTions in Patients with Severe Brain Injury – an InterVEntional Cluster Randomized Trial

(DETECT-IVE)

Version 1.0

July 10, 2025

Prof. Dr. Kristian Barlinn
Principal investigator
Department of Neurology; Dresden Neurovascular Center
University Hospital Carl Gustav Carus and TU Dresden
Dresden, Germany

Dresden; July 10, 2025;

Place; date; signature

Dr. Martin Roessler
Responsible statistician
Department of Neurology; Dresden Neurovascular Center
University Hospital Carl Gustav Carus and TU Dresden
Dresden, Germany and
BARMER Institute for Health Care System Research (BARMER Institut für Gesundheitssystemforschung; bifg)
Berlin, Germany

Berlin; July 10, 2025

Place; date; signature

Contents

Objectives and hypotheses 4
Study objectives 4
Hypotheses 4
Study framework 4
Intervention 4
Participating hospitals 5
Trial design 5
Randomization 6
Data 7
Case and variable definitions 8
Inclusion and exclusion criteria 8
Outcomes 8
Primary outcome 8
Secondary outcomes 8
Intervention 8
Confounders 9
Intention-to-treat and per-protocol analysis 9
Handling of missing and implausible values 10
Missing values 10
Drop out/lost to follow-up 10
Implausible values 10
Statistical analyses 10
Descriptive analysis 10
Significance and confidence levels 10
Main analysis (primary outcome) 11
Analyses of secondary outcomes 12
Sensitivity analyses 12
Adjustment for potential confounders 12
Alternative technical specification of the estimation procedure 12
Potential effect modifications 12
Effect modification by the previous share of detected brain death 12
Effect modification by university hospital status 12
Effect modification by different utilizations of DETECT 13

Statistical software 13
References.. 13

Abbreviations

ICU	Intensive care unit
DETECT	AutomateD ScrEening for Clinically AscerTainEd Loss of Cerebral FuncTions in Patients with Severe Brain Injury
DSO	Deutsche Stiftung Organtransplantation
SW-CRT	Stepped wedge cluster randomized trial
MICE	Multiple imputation by chained equations

Objectives and hypotheses

Study objectives

The primary objective of the DETECT-IVE study is to evaluate the effectiveness of the Automated Screening for Clinically Ascertained Loss of Cerebral Functions in Patients with Severe Brain Injury tool (DETECT) regarding the detection of patients with irreversible loss of brain function (brain death).

The secondary objectives of the DETECT-IVE study are to explore relationships between the implementation of DETECT and 1) missed patients with potential brain death, 2) organ donation, and 3) donation-related interactions with the German organ procurement organization (Deutsche Stiftung Organtransplantation; DSO).

Hypotheses

The primary hypothesis is that the implementation of DETECT causally increases the probability of detecting patients with brain death in hospitals without prior use of DETECT.

Secondary hypotheses are that the implementation of DETECT is related to

- a lower probability of missing patients with potential brain death,
- a higher probability of organ donation, and
- a higher probability of donation-related interactions with the DSO

in hospitals without prior use of DETECT.

Study framework

Intervention

The intervention is the implementation of DETECT in the hospitals participating in this study. DETECT was initiated at University Hospital Carl Gustav Carus Dresden and further developed in collaboration with the Data Integration Center (DIZ) of University Hospital Carl Gustav Carus Dresden and the DSO. DETECT periodically screens data entered manually into the patient data management system (PDMS) according to defined criteria and creates a results list of detected cases with potential brain death. The defined criteria for screening include clinical findings such as coma, which is quantified using the Richmond Agitation Sedation Scale (RASS) or the Glasgow Coma Scale (GCS), as well as the extinguished light reaction of the pupils (so-called “hard indicators”). These findings can indicate an impending loss of overall brain function at an early stage. Additional information (so-called “soft indicators”) such as the serum sodium level, ventilation mode, intracranial pressure (ICP), cerebral perfusion pressure (CPP) and the indication of resuscitation are not primarily used by DETECT for the direct assessment of the patient's condition but provide the transplant officer with additional information if hard indicators are already fulfilled.

In the event of detection of a potential case with brain death, a notification is sent automatically via the hospital's internal email server to the transplant officer or to a group of people individually defined for each intensive care unit (ICU). This notification contains the above-mentioned patient information and is intended to support standardized processes. For instance, it can induce an individual case evaluation of the detected patient in the ICU by the treating ICU staff. In the case of a clinically confirmed critical constellation of findings, it may result in an indication check for guideline-compliant diagnostics of brain death.

Participating hospitals

In total, 19 hospitals located in different German regions were included in the study (Table 1). Each of these hospitals had at least one ICU treating patients potentially fulfilling the inclusion criteria at the start of the study. Eleven of the hospitals were university hospitals. DETECT was not previously used in the participating hospitals and, thus, the first implementation of DETECT was planned within the framework of this study.

Table 1: Participating hospitals

Hospital name	University hospital
Charité Universitätsmedizin Berlin Campus Benjamin Franklin	yes
Charité Universitätsmedizin Berlin Campus Charité Mitte	yes
Charité Universitätsmedizin Berlin Campus Virchow	yes
Elblandklinikum Meißen	no
Elblandklinikum Radebeul	no
Elblandklinikum Riesa	no
Heinrich-Braun-Klinikum Zwickau	no
Klinikum Altenburger Land GmbH	no
Klinikum Chemnitz	no
Klinikum St. Georg Leipzig	no
Universitätsklinikum Aachen	yes
Universitätsklinikum Bochum	yes
Universitätsklinikum Bonn	yes
Universitätsklinikum Göttingen	yes
Universitätsklinikum Halle	yes
Universitätsklinikum Jena	yes
Universitätsklinikum Magdeburg	yes
Universitätsklinikum Mainz	yes
Zentralklinik Bad Berka	no

Trial design

The trail was designed as a stepped wedge cluster randomized trial (SW-CRT) [1]. Before the start of the study, the 19 participating hospitals were randomly assigned to different, pre-defined intervention steps within a 30-months study period (Figure 1). At the start of the study, DETECT was not activated in any hospital (control phase). The first hospital was planned to enter the intervention phase in study

month 2 by activating DETECT. Since the start of the study was on February 15, 2025, the first hospital was planned to enter the intervention phase on March 15, 2025. After entering the intervention phase, each hospital was expected to continuously use DETECT throughout the rest of the study period.

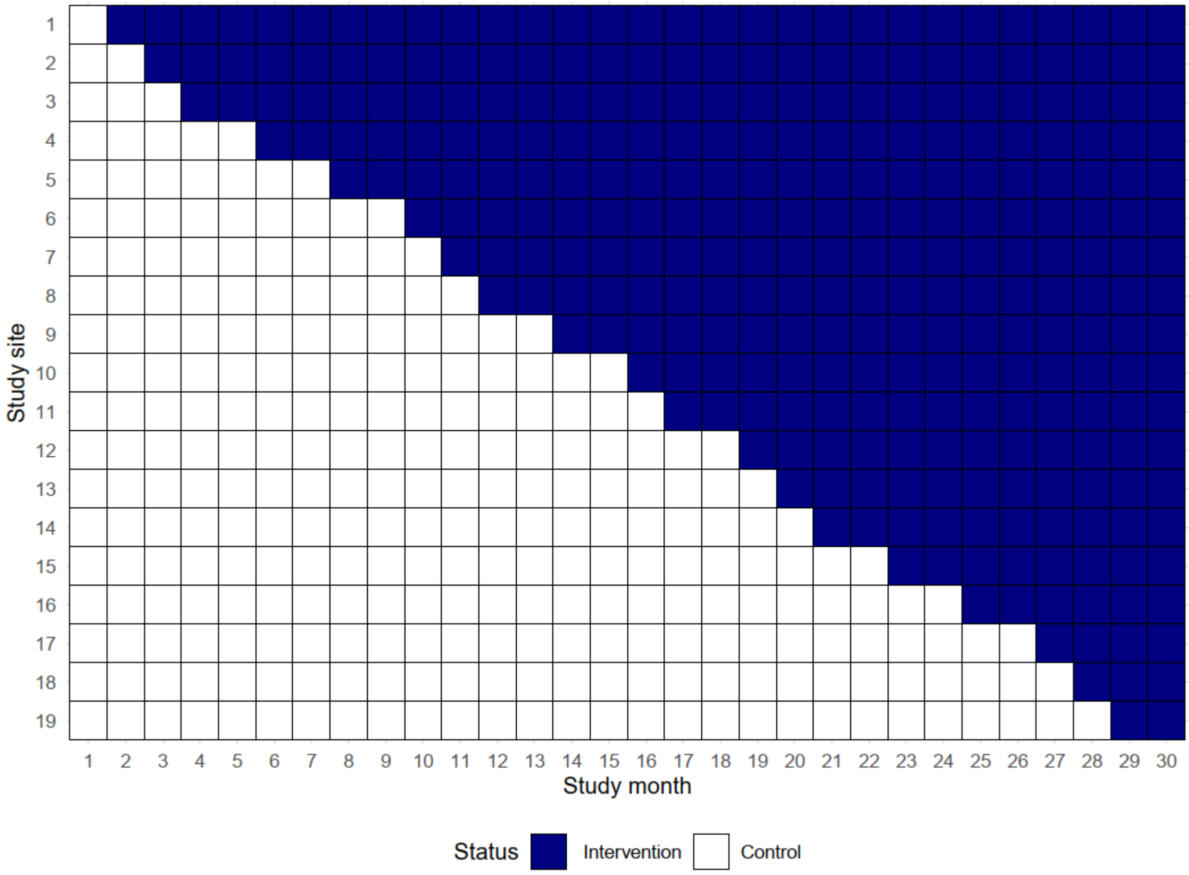

Figure 1: Intervention and control phases for the participating hospitals within the study period

Randomization

Hospitals were randomly assigned to the intervention steps (Figure 1) before the start of the study. For this purpose, hospitals were first sorted alphabetically by name. In a second step, a random number drawn from a continuous uniform distribution with boundaries 0 and 1 was assigned to each hospital. Hospitals were then sorted according to these numbers from lowest (intervention step 1) to highest (intervention step 19) to derive the intervention order and the corresponding intervention dates (Table 2). Randomization was conducted only once by the responsible statistician. A fixed seed was used for reproducibility.

Table 2: Intervention order and intervention dates

Intervention step	Intervention date	Hospital name
1	2025-03-15	Universitätsklinikum Mainz
2	2025-04-15	Klinikum Altenburger Land GmbH
3	2025-05-15	Universitätsklinikum Göttingen
4	2025-07-15	Charité Universitätsmedizin Berlin Campus Virchow
5	2025-09-15	Universitätsklinikum Aachen
6	2025-11-15	Universitätsklinikum Halle
7	2025-12-15	Zentralklinik Bad Berka
8	2026-01-15	Elblandklinikum Riesa
9	2026-03-15	Elblandklinikum Radebeul
10	2026-05-15	Klinikum Chemnitz
11	2026-06-15	Klinikum St. Georg Leipzig
12	2026-08-15	Universitätsklinikum Bochum
13	2026-09-15	Heinrich-Braun-Klinikum Zwickau
14	2026-10-15	Elblandklinikum Meißen
15	2026-12-15	Charité Universitätsmedizin Berlin Campus Benjamin Franklin
16	2027-02-15	Universitätsklinikum Bonn
17	2027-04-15	Charité Universitätsmedizin Berlin Campus Charité Mitte
18	2027-05-15	Universitätsklinikum Jena
19	2027-06-15	Universitätsklinikum Magdeburg

Data

The analysis will be based on anonymized patient data collected according to the German Organ Transplantation Act (§ 9a Abs. 2 Gesetz über die Spende, Entnahme und Übertragung von Organen und Geweben (TPG)). These data contain all patients with primary or secondary brain injury who died in the respective hospital. The data include information on age, sex, type of brain injury (primary/secondary), ventilation status and duration, length of stay (total and on ICU), diagnoses coded according to the International Statistical Classification of Diseases and Related Health Problems, 10th revision, German Modification (ICD-10-GM), detected brain death, and organ donation. Complete reporting of these data to the DSO is obligatory for all German hospitals.

In addition, the physicians acting as transplant officers in the hospitals routinely screen patient data for potentially undetected patients with brain death using the software "DSO-TransplantCheck 4" every six months. The results of these screenings are used to operationalize the number of missed cases with potential brain death. Within the framework of this study, the screenings of all participating hospitals will be supported by a study physician to ensure homogeneous processes and comparable results.

We will complement these patient data with information on the use of DETECT after implementation in the participating hospitals. This includes functionality, recording and documentation of clinical

findings, generation of e-mails, and time stamps. These data will be used within the framework of sensitivity analysis to explore potential heterogeneity in the effects of DETECT on the primary and secondary outcomes (see below).

Case and variable definitions

Inclusion and exclusion criteria

The study population encompasses all patients with potential brain death. This includes patients

- with primary and/or secondary brain injury,
- that were invasively ventilated during their hospital stay
- and were discharged because of in-hospital death.

We will exclude all patients under 18 years of age at the time of hospital admission.

Outcomes

Primary outcome

The primary outcome of the study is the detection of patients with brain death. The definition of brain death adheres to the corresponding guideline of the German Medical Association (Bundesärztekammer) [2]. Detection of brain death is recorded in the data provided by the hospitals to DSO according to TPG (see section "Data"). Detection of brain death will be represented by a binary variable (0=no brain death detected; 1= brain death detected) for each patient included in the statistical analysis.

Secondary outcomes

We consider the following secondary outcomes, which may be related to the use of DETECT:

- missed case with potential brain death (0=no; 1=yes),
- successful organ donation (0=no; 1=yes),
- donation-related interaction with the DSO (0=no; 1=yes),
- number of alerts sent by DETECT.

All these outcomes will be analyzed at the patient level, at the hospital level, and by intervention phase.

Intervention

The intervention is the implementation and use of DETECT by the hospital. A patient will be considered as having received the intervention if DETECT was implemented in the treating hospital before the patient was discharged because of in-hospital death. The intervention will be represented by an indicator variable (0=patient did not receive the intervention; 1=patient received the intervention) in statistical analyses.

Confounders

Potential confounders at the patient level considered in sensitivity analyses include

- age (in years and in categories),
- sex (male/female),
- type of brain injury (primary/secondary),
- selected diseases as coded by primary diagnoses according to the International Statistical Classification Of Diseases And Related Health Problems, 10th revision, German Modification (ICD-10-GM):
    - Traumatic brain injury (ICD-10-GM: S06),
    - Ischemic Stroke (ICD-10-GM: I63),
    - Intracerebral hemorrhage (ICD-10-GM: I61) ,
    - Subarachnoid hemorrhage (ICD-10-GM: I60),
    - Postanoxic brain injury (e.g., after cardiac arrest) (ICD-10-GM: G93.1),
    - Central nervous system infection (ICD-10-GM: G00; G04),
    - Other causes (e.g., tumor) (ICD-10-GM: C*).

If additional or alternative ICD-10-GM codes are present, they will be assigned to the above categories if they match one of the defined etiologies. ICD-10-GM codes that cannot be clearly attributed to a specific underlying etiology (e.g., G93.6 cerebral edema or G93.5 brain compression) will be assigned to the disease category "Other".

At the hospital level, potential confounders include

- the number of patients fulfilling the inclusion criteria and not fulfilling the exclusion criteria within one year before the start of the study,
- the share of detected brain death in the one-year period before the start of the study,
- university hospital status (0=other hospital; 1=university hospital).

The first two of these potential hospital-level confounders are used to operationalize the experience of a hospital in the treatment of patients belonging to the study population and in the detection of brain death.

Intention-to-treat and per-protocol analysis

The main analysis will be based on the intention-to-treat (ITT) principle. In particular, a patient will be considered as having received the intervention if he/she was discharged at or after the intervention date assigned to the hospital, regardless of the actual implementation status of DETECT in that hospital. ITT analysis generally results in conservative intervention effect estimates [3].

As a complementary approach, we will apply per-protocol (PP) analysis [3]. If a hospital does not implement DETECT at its assigned intervention date but with delay, patients dying in that hospital in the period between the assigned intervention date and the actual intervention date will be excluded in PP analysis.

Handling of missing and implausible values

Missing values

Missing values will be addressed using multiple imputation by chained equations (MICE) [4]. As an alternative approach, we will additionally apply complete case analysis by excluding all patients with missing values in at least one variable entering the respective statistical model.

Drop out/lost to follow-up

In general, a drop-out or lost-to-follow-up of patients is not possible according to the definition of the study population. A drop-out of a hospital is possible if the hospital a) voluntarily withdraws from the study within the study period, b) changes its portfolio of medical services such that it does no longer treat patients belonging to the study population, c) is closed within the study period. In each case, the main analysis will include the data provided by such hospitals until the time of drop out. For sensitivity analysis, the complete data of hospitals with drop-out will be removed from the sample.

Implausible values

Implausible values may be detected by descriptive analysis of the data. Such values will undergo clinical judgement by clinical experts in the study team. If possible, implausible values will be corrected. Otherwise, they will be treated as missing values (see above). The handling of all implausible values will be documented and published along with the study results.

Statistical analyses

Descriptive analysis

Categorical variables will be described by absolute and relative frequencies. The distribution of continuous variables will be described by median, mean, interquartile range (IQR), minimum and maximum values. For each hospital, we will visualize the shares of primary and secondary outcomes over time. Distributions of variables and development of indicators over time will be depicted by suitable diagrams such as bar charts, box plots, violin plots, line plots, and scatter plots. Descriptive statistics will generally be reported by intervention status (control phase / intervention phase).

Significance and confidence levels

We will apply a significance level of 5% for all statistical tests. Statistical estimates of parameters will be reported with 95%-confidence intervals. If required, we will account for intra-cluster correlation and conduct appropriate corrections of degrees of freedom when calculating p-values and confidence intervals (see below).

Main analysis (primary outcome)

The main analysis of the primary outcome will be based on a generalized linear mixed model (GLMM). Adapting the specification proposed by Hussey and Hughes [1], we will model the expected value of the primary outcome $Y_{hti_{ht}}$ ($Y_{hti_{ht}} = 0$: brain death was not detected; $Y_{hti_{ht}} = 1$: brain death was detected) of individual $i_{ht} = 1, \ldots, n_{ht}$ treated in hospital $h = 1, \ldots, H$ in study month $t = 1, \ldots, T$ as

$$E\left[Y_{hti_{ht}} \middle| \alpha_h, \beta_t, D_{ht}\right] = F^{-1}(\mu + \alpha_h + \beta_t + \theta D_{ht}),$$

where

- $F(\cdot)$ is the logistic link function,
- μ is a constant,
- $\alpha_h \overset{i.i.d.}{\sim} N(0, \sigma^2)$ is a normally distributed, hospital-specific random effect with variance σ^2,
- β_t represents time fixed effects (estimated by inclusion of $T - 1$ time dummies in the regression model),
- D_{ht} is a binary treatment indicator for hospital h at study month t ($D_{ht} = 0$: intervention has not yet been implemented, $D_{ht} = 1$: intervention has already been implemented),
- θ is the intervention effect.

Due to the inclusion of α_h, this model captures correlation of patient outcomes within hospitals (i.e. intra-cluster correlation). The estimation of β_t by inclusion of time dummies in the model accounts for potential confounding by unobserved time effects.
For interpretability, the intervention effect θ will be transformed into an odds ratio by $\mathrm{OR}(\theta) = \exp(\theta)$, which reflects the chance of brain death detection in the intervention phase relative to the control phase. In line with the primary hypothesis, we expect that $\mathrm{OR}(\theta) > 1$, indicating that the intervention leads to a higher chance of detecting brain death.

Conventional approaches to statistical inference based on mixed effects models in cluster randomized trials may be invalid when the number of clusters is small. In particular, a small number of clusters is likely to induce an inflated type I error rate [5]. Against that background, we will follow recent recommendations in the methodological literature [6] by 1) using restricted pseudo-likelihood estimation and 2) deriving the p-value and the confidence interval of the intervention effect based on a t-distribution with $H - K$ degrees of freedom, where H is the number of hospitals included in the analysis and K is the number of estimated cluster-level parameters. Accordingly, we will use a two-sided t-test based on a t-distribution with $H - 2$ degrees of freedom in the main analysis.

The model specification and inference approach that will be used for the main analysis was also used for power calculation before the start of the study (see the Appendix of the statistical analysis plan). This calculation yielded an expected power of 83.6 %.

Analyses of secondary outcomes

The analysis of secondary outcomes will be based on GLMMs analogous to the model specified for the primary outcome. For the binary outcomes “missed case with potential brain death”, “successful organ donation”, and “donation-related interaction with the DSO” a logistic link function will be used. For the outcome “number of alerts sent by DETECT”, we will apply a log-link function. If required, we will use robust standard error estimators to account for overdispersion.

Sensitivity analyses

Sensitivity analyses will be conducted for the effect of the intervention on the primary and secondary outcomes as described below.

Adjustment for potential confounders

We will address potential imbalances between control and intervention phase regarding the above-mentioned confounders by inclusion of these confounders as regressors in the statistical models. Each potential confounder will be included a) as a single additional regressor and b) together with all other potential confounders in separate model specifications.

Alternative technical specification of the estimation procedure

To investigate the sensitivity of statistical results regarding technical specifications of the estimation procedure, we will estimate additional GLMMs with adaptive quadrature and without correction of degrees of freedom. The link functions and the specifications of the linear predictor will be similar to the main specifications described above.

Potential effect modifications

Potential effect modifications will be explored with respect to the relationships between the intervention and the primary and secondary outcomes as described below.

Effect modification by the previous share of detected **brain death**

Hospitals with low initial shares of detected patients with brain death may exhibit higher potential for improvement of detection rates than hospitals with high initial shares of detected patients with brain death. To test this hypothesis, we will use the hospitals’ shares of detected patients with brain death one year prior to the start of this study as a potential moderator variable. This moderator variable will enter the statistical models for the primary and the secondary outcomes as a multiplicative interaction term with the binary treatment indicator. In addition, we will conduct stratified estimations by splitting hospitals into two samples according to the median value of the initial shares of detected patients with brain death.

Effect modification by university hospital status

In another analysis, we will investigate whether university hospitals differ from other hospitals in terms of the effectiveness of DETECT. To explore this potential effect heterogeneity, we will include a

multiplicative interaction term between university hospital status (0=no; 1=yes) and the binary treatment indicator into the statistical models. In addition, we will estimate separate models for university hospitals and other hospitals.

Effect modification by different utilizations of DETECT

The effectiveness of DETECT may depend on the type and the intensity of the utilization of DETECT. To operationalize variations in utilization, we will consider different potential moderator variables:

- the use of DETECT for recording and communication of patient-specific information by the physicians (0=no; 1=yes),
- the number of (patient-specific and/or time-period-specific) alerts send by DETECT,
- the use of a built-in question feature that allows communication among clinical staff regarding the patient cases.

Each of these moderator variables will enter the statistical models for the primary and secondary outcomes as a multiplicative interaction term with the binary treatment indicator. In addition, stratified estimations will be conducted by splitting the sample according to the categories of categorical moderator variables and median values of continuous moderator variables, respectively.

Statistical software

For data preparation, we will use SQL and Excel. Statistical analysis will be conducted using R (version 4.5.0).

References

1 Hussey MA, Hughes JP. Design and analysis of stepped wedge cluster randomized trials. *Contemporary Clinical Trials*. 2007;28:182–91. doi: 10.1016/j.cct.2006.05.007

2 Bundesärztekammer. Richtlinie gemäß § 16 Abs. 1 S. 1 Nr. 1 TPG für die Regeln zur Feststellung des Todes nach § 3 Abs. 1 S. 1 Nr. 2 TPG und die Verfahrensregeln zur Feststellung des endgültigen, nicht behebbaren Ausfalls der Gesamtfunktion des Großhirns, des Kleinhirns und des Hirnstamms nach § 3 Abs. 2 Nr. 2 TPG, Fünfte Fortschreibung. 2022.

3 Gupta SK. Intention-to-treat concept: A review. *Perspectives in Clinical Research*. 2011;2:109–12. doi: 10.4103/2229-3485.83221

4 Azur MJ, Stuart EA, Frangakis C, *et al.* Multiple imputation by chained equations: what is it and how does it work? *International Journal of Methods in Psychiatric Research*. 2011;20:40–9. doi: 10.1002/mpr.329

5 Leyrat C, Morgan KE, Leurent B, *et al.* Cluster randomized trials with a small number of clusters: which analyses should be used? *International Journal of Epidemiology*. 2018;47:321–31. doi: 10.1093/ije/dyx169

6 Thompson JA, Leyrat C, Fielding KL, *et al.* Cluster randomised trials with a binary outcome and a small number of clusters: comparison of individual and cluster level analysis method. *BMC Medical Research Methodology*. 2022;22:222. doi: 10.1186/s12874-022-01699-2
